# The minimal COVID-19 vaccination coverage and efficacy to compensate for a potential increase of transmission contacts, and increased transmission probability of the emerging strains

**DOI:** 10.1186/s12889-022-13429-w

**Published:** 2022-06-27

**Authors:** Biao Tang, Xue Zhang, Qian Li, Nicola Luigi Bragazzi, Dasantila Golemi-Kotra, Jianhong Wu

**Affiliations:** 1 School of Mathematics and Statistics, Xi’an Jiaotong University, Xi’an, China; 2 The Interdisciplinary Research Center for Mathematics and Life Sciences, Xi’an Jiaotong University, Xi’an, China; 3 Department of Mathematics, Northeastern University, Shenyang, Liaoning China; 4 Laboratory for Industrial and Applied Mathematics, York University, Toronto, Ontario Canada; 5 Department of Biology, York University, Toronto, Ontario Canada; 6 Laboratory of Mathematics for Public Health, Fields Institute, Toronto, Ontario Canada

**Keywords:** COVID-19, Vaccination, Compartment model, Reproduction numbers, Final Size, Attack Rate, Strain variations

## Abstract

**Background:**

Mass immunization is a potentially effective approach to finally control the local outbreak and global spread of the COVID-19 pandemic. However, it can also lead to undesirable outcomes if mass vaccination results in increased transmission of effective contacts and relaxation of other public health interventions due to the perceived immunity from the vaccine.

**Methods:**

We designed a mathematical model of COVID-19 transmission dynamics that takes into consideration the epidemiological status, public health intervention status (quarantined/isolated), immunity status of the population, and strain variations. Comparing the control reproduction numbers and the final epidemic sizes (attack rate) in the cases with and without vaccination, we quantified some key factors determining when vaccination in the population is beneficial for preventing and controlling future outbreaks.

**Results:**

Our analyses predicted that there is a critical (minimal) vaccine efficacy rate (or a critical quarantine rate) below which the control reproduction number with vaccination is higher than that without vaccination, and the final attack rate in the population is also higher with the vaccination. We also predicted the worst case scenario occurs when a high vaccine coverage rate is achieved for a vaccine with a lower efficacy rate and when the vaccines increase the transmission efficient contacts.

**Conclusions:**

The analyses show that an immunization program with a vaccine efficacy rate below the predicted critical values will not be as effective as simply investing in the contact tracing/quarantine/isolation implementation. We reached similar conclusions by considering the final epidemic size (or attack rates). This research then highlights the importance of monitoring the impact on transmissibility and vaccine efficacy of emerging strains.

## Background

The “Severe Acute Respiratory Syndrome-related Coronavirus type 2” (SARS-CoV-2) has caused a global pandemic since it was first reported in the Wuhan city of the Hubei Province of China, in December of 2019 [1]. As of December 26, 2020, there were 78,383,527 COVID-19 confirmed cases and 1,740,390 death cases linked to COVID-19 globally [2], which have become 466,733,118 infections and 6,089,484 deaths, respectively, as of March 18, 2022. It remains paramount to circumvent the spread of COVID-19, especially considering the emergence of strain variation and its impact on transmissibility.

Vaccination, as an important preventative method, has substantially improved health and reduced mortality outcomes for many infectious diseases [3, 4]. There have been significant COVID-19 vaccine developments that mass vaccination in the population in the first half of 2021 has become possible and this immunization can be potentially the most effective method to successfully control the local outbreaks and global spread of the COVID-19 pandemic.

Several modeling studies have attempted to analyze the role of vaccination in controlling COVID-19 epidemics [5–7], especially with a focus on optimal vaccination programs [8–12]. There remain great challenges in using vaccination to mitigate the COVID-19 epidemics as vaccination in the population could potentially lead to an increase in the transmission contacts due to the perceived vaccine-provided immunity. Specifically, when a vaccine is used in a portion of the population to mitigate COVID-19 transmission, due to the perceived vaccine-provided immunity, the population may 1) increase their contact levels with increasing social-economic activities, and/or reduce their personal protection (less physical distancing and mask-wearing); 2) not quarantine themselves even if their close contacts with infected individuals have been identified through contact tracing, and 3) not isolate themselves during the pre-symptomatic stage of the infectious period or during their asymptomatic infection period when the vaccine fails to provide them with protection against the infection.

Besides risk perception, further important factors that could impact the outcomes of vaccination campaigns are given by public health policies and how they shape the unfolding of vaccine roll-out and uptake. While some policies can have beneficial effects, by targeting vaccine hesitancy and empowering the communities, other policies, including the so-called “vaccine nationalism” [13], may result in detrimental impacts, by introducing vaccine inequity and seriously jeopardizing the implementation of public health measures, especially in low-and-middle-income countries (LMICs) [14–17]. However, since the present study is based on the local (rather than global) level, these latter parameters will not be incorporated in the present study and warrant further *ad hoc* research. Moreover, the present study will not consider other potential (co-)infections or interactions of COVID-19 with emerging/re-emerging diseases [18–27]. Once again, this warrants separate, specific studies.

The main purpose of this study is to use a mathematical model to quantify the minimum vaccination coverage and vaccine efficacy to compensate for a potential increase in transmission contacts, and decrease in personal protection and/or in compliance with quarantine and isolation protocol when identified as close contacts of infections. The article is organized as follows: the mathematical model is formulated in the Methods section and is used to examine the impact of vaccination on the control reproduction numbers as well as the final epidemic size in the Results section. We then draw conclusions and mark important points of our study in the Discussion section.

## Methods

We designed a mathematical model of COVID-19 transmission dynamics in the population by assuming that a portion of the population was vaccinated against COVID-19 to mitigate the COVID-19 transmission. The transmission model is based on those published and tested against the real data [28–32].

**Fig. 1:**
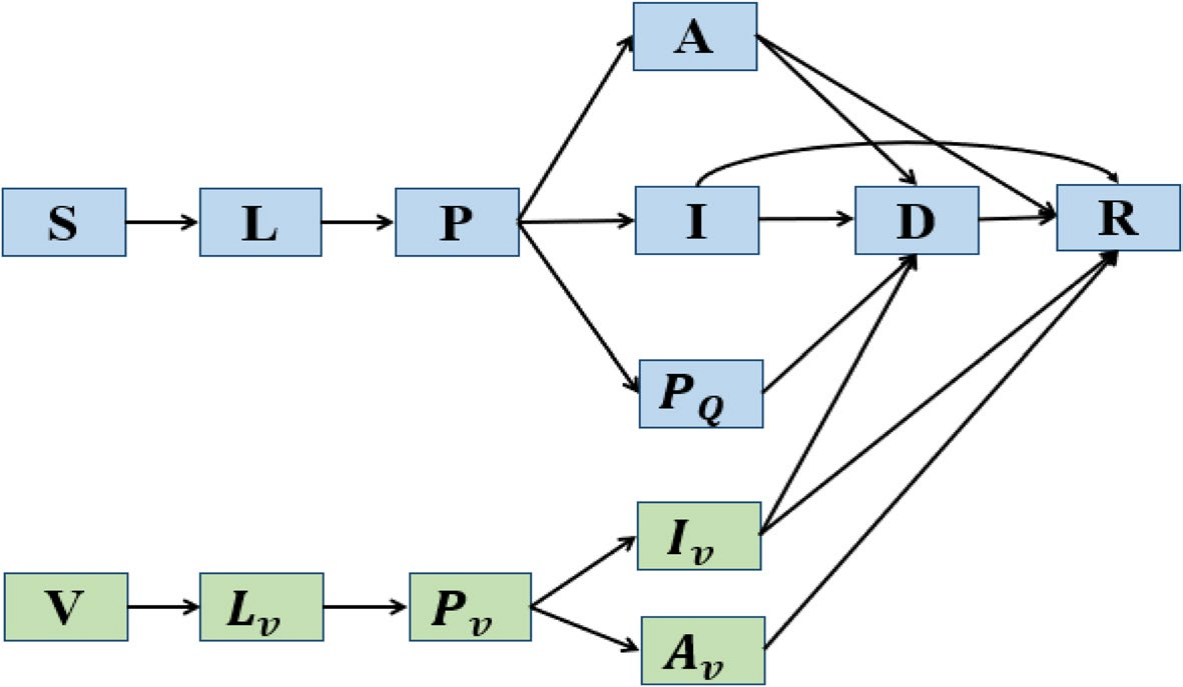
An illustration of the COVID-19 transmission dynamics, when the population is stratified by the epidemiological status, public health status (quarantined/isolated) and immunization status

We use *v* for the vaccination coverage. Then the portion (1 − *v*) of the total population is the population without vaccination, and this unvaccinated population is divided into susceptible (*S*), exposed (*L*), pre-symptomatic (*P*), symptomatic infectious (*I*), asymptomatic infectious (*A*), and recovered (*R*) compartments according to the epidemiological status of individuals. This population is divided further into diagnosed and isolated (*D*) and quarantined (*P*_*Q*_) compartments according to the public health intervention status of individuals (Fig. 1). More precisely, we assume that a proportion, *q*, of COVID-19 infected individuals can be traced and quarantined, while the other proportion, 1 − *q*, will either move to A class or I class depending on whether they show symptoms. The ratio of asymptomatic infections is assumed to be *ξ*.

The rest of the population, with a ratio *v*, will be vaccinated against COVID-19. Further, we assume that the vaccine efficacy is *η*. Therefore, the proportion, *vη*, will have the immunity to COVID-19, while the other proportion, *v*(1 − *η*), will remain susceptible to COVID-19 even after they are vaccinated (Table 1). Similarly, we divide this population (i.e. the portion of population vaccinated but still susceptible to COVID-19) into susceptible (*V*), exposed (*L*_*v*_), pre-symptomatic (*P*_*v*_), symptomatic infectious (*I*_*v*_), asymptomatic infectious (*A*_*v*_) compartments. We also denote the diagnosed and recovered population with vaccination as *D* and *R*, respectively. As for the vaccinated population, we assume that they will not be traced and quarantined.

**Table 1:** Definitions and values of parameters

Parameter	Definitions	Value	Source
*β*	Transmission rate for non-vaccines	Vary	[28]
*β*_*v*_	Transmission rate for vaccines	Vary	[28]
*v*	Vaccination coverage of COVID-19	Vary	Assumed
*η*	Efficacy rate of COVID-19 vaccine	Vary	Assumed
*θ*_1_	Modification factor of pre-symptomatic infectiousness	0.0275	Assumed
*θ*	Modification factor of asymptomatic infectiousness	0.0275	[28]
*σ*	Transition rate of exposed individuals without vaccination to pre-symptomatic class	1/3	[32]
*σ*_*v*_	Transition rate of exposed individuals with vaccination to pre-symptomatic class	1/3	[32]
1/ρ	Pre-symptomatic period	1/2	[32]
*q*	Quarantine fraction	Vary	[28]
*ξ*	Probability of having no symptom among infected individuals	0.2964	[28]
*δ*	Transition rate of the symptomatic infected individual without vaccination to the diagnosed and quarantined infected class	0.1344	[28]
*δ*_*Q*_	Transition rate of the quarantined infected individuals without vaccination to the diagnosed and quarantined infected class	0.1237	[28]
*δ*_*A*_	Transition rate of the asymptomatic infected individuals without vaccination to the diagnosed and quarantined class	0.1237	Assumed
*δ*_*v*_	Transition rate of the symptomatic infected individuals with vaccination to the diagnosed and quarantined class	0.1344	Assumed
*γ*	Recover rate of the symptomatic infected individuals without vaccination	0.1957	[28]
*γ*_*v*_	Recover rate of the symptomatic infected individuals with vaccination	0.1957	Assumed
*γ*_*Av*_	Recovery rate of asymptomatic infected individuals with vaccination	0.139	Assumed
*γ*_*A*_	Recovery rate of asymptomatic infected individuals without vaccination	0.139	[28]
*γ*_*D*_	Recovery rate of quarantined diagnosed individuals	0.2	[28]
*α*	Disease-induced death rate	0.008	[28]

We note that most vaccine candidates approved require administration of two-doses to achieve the maximal efficacy. Because of the supply constraint, vaccines will also not be delivered and administrated in the entire population at the same time. These logistic constraints and their implications for the limitation of our study will be addressed in the final Discussion section.

The transmission diagram is shown in Fig. 1, and the corresponding compartmental model is as follows:

1 $$\left\{\begin{array}{c}{S}^{'}=-\frac{\beta \left(I+\theta A+{I}_v+\theta {A}_v+{\theta}_1P+{\theta}_1{P}_v\right)S}{N},\kern5.25em \\ {}{L}^{'}=\frac{\beta \left(I+\theta A+{I}_v+\theta {A}_v+{\theta}_1P+{\theta}_1{P}_v\right)S}{N}-\sigma L,\kern3.5em \\ {}{P}^{'}=\sigma L-\rho P,\kern13.5em \\ {}{P_Q}^{'}= q\rho P-{\delta}_Q{P}_Q,\kern11.5em \\ {}{I}^{'}=\left(1-\xi \right)\left(1-q\right)\rho P-\gamma I-\delta I,\kern5em \\ {}{A}^{'}=\xi \left(1-q\right)\rho P-{\delta}_AA-{\gamma}_AA,\kern6.25em \\ {}{V}^{'}=-\frac{\beta_v\left(I+\theta A+{I}_v+\theta {A}_v+{\theta}_1P+{\theta}_1{P}_v\right)V}{N},\kern5em \\ {}{L_v}^{'}=\frac{\beta_v\left(I+\theta A+{I}_v+\theta {A}_v+{\theta}_1P+{\theta}_1{P}_v\right)V}{N}-{\sigma}_v{L}_v,\kern2.25em \\ {}{P_v}^{'}={\sigma}_v{L}_v-\rho {P}_v,\kern12em \\ {}{I_v}^{'}=\left(1-\xi \right)\rho {P}_v-{\gamma}_v{I}_v-{\delta}_v{I}_v,\kern5.5em \\ {}{A_v}^{'}=\xi \rho {P}_v-{\gamma}_{Av}{A}_v,\kern10.5em \\ {}{D}^{'}={\delta}_Q{P}_Q+\delta I+{\delta}_AA+{\delta}_v{I}_v-{\gamma}_DD-\alpha D,\\ {}{R}^{'}=\gamma I+{\gamma}_AA+{\gamma}_v{I}_v+{\gamma}_{Av}{A}_v+{\gamma}_DD.\kern2.5em \end{array}\right.$$

In the above formulation, *N* is the total population, that is,

$$N=S+L+P+A+I+{P}_Q+V+{L}_v+{P}_v+{I}_v+{A}_v+D+R.$$

We use *β* and *β*_*v*_ to denote the transmission rates of infectious individuals with and without vaccination, respectively. These rates, *β* and *β*_*v*_, are the *disease transmission effective contacts* (per day), defined as the contacts (per day) multiplied by the transmission probability per contact. Therefore, the increase of disease transmission effective contacts can result from the increase of social economical activities, the decrease of personal protection, or a combination of both. Therefore, *β*_*v*_ > *β* can happen if the vaccinated individuals have increased effective contacts.

The detailed definitions of all the other parameters are listed in Table 1.

We will describe our results using the so-called control reproduction number. This is the total number of new infections generated by an infective individual, it is called control reproduction number since our model reflects the realty that certain control interventions are already in place. It is well known that an outbreak can be prevented if the control reproduction number is below the threshold value 1. It is also known that if an outbreak cannot be prevented by the control interventions, then the larger the control reproduction number, the large the exponential growth rate of the outbreak. Therefore, in what follows, we examine when the control reproduction number can be less than the threshold value (with and/or without vaccination), and compare the value of the control reproduction numbers with and without vaccination.

We first derive the formula for the control reproduction numbers: the control reproduction number ${R}_0^v$ when vaccination is used, and the control reproduction number *R*_0_ when the vaccine is not used in the population. Following the standard next generation approach [31]. These can be calculated explicitly in terms of model parameters and initial conditions:

Control reproduction number with vaccination:

$${\displaystyle \begin{array}{l}{R}_0^v=\frac{\beta {S}_0}{N_0}\left(\frac{\theta_1}{\rho }+\frac{\left(1-\xi \right)\left(1-q\right)}{\gamma +\delta }+\frac{\theta \xi \left(1-q\right)}{\delta_A+{\gamma}_A}\right)+\frac{\beta_v{V}_0}{N_0}\left(\frac{\theta_1}{\rho }+\frac{\left(1-\xi \right)}{\gamma_v+{\delta}_v}+\frac{\theta \xi}{\gamma_{Av}}\right)\\ {}=\beta \left(1-v\right)\left(\frac{\theta_1}{\rho }+\frac{\left(1-\xi \right)\left(1-q\right)}{\gamma +\delta }+\frac{\theta \xi \left(1-q\right)}{\delta_A+{\gamma}_A}\right)+{\beta}_v\left(1-\eta \right)v\left(\frac{\theta_1}{\rho }+\frac{\left(1-\xi \right)}{\gamma_v+{\delta}_v}+\frac{\theta \xi}{\gamma_{Av}}\right),\end{array}}$$

where, *S*_0_ = (1 − *v*)*N*_0_, *V*_0_ = (1 − *η*)*vN*_0_ are the initial susceptible population without vaccination and with vaccination, respectively, *N*_0_ is initial total population.

Control reproduction number without vaccination:

$${R}_0=\beta \left(\frac{\theta_1}{\rho }+\frac{\left(1-\xi \right)\left(1-q\right)}{\gamma +\delta }+\frac{\theta \xi \left(1-q\right)}{\delta_A+{\gamma}_A}\right)$$

We will also link the control reproduction number to the final size (the attack rate) in the next section.

## Results

### R1. Impact of mass vaccination on control reproduction numbers

We first focus on comparing the two control reproduction numbers to evaluate when vaccination in the population is beneficial for the control of a future COVID-19 outbreak.

Let ${R}_d={R}_0^v-{R}_0$. Then, we obtain:

$${R}_d=v\left[{\beta}_v\left(1-\eta \right)\left(\frac{\theta_1}{\rho }+\frac{\left(1-\xi \right)}{\gamma_v+{\delta}_v}+\frac{\theta \xi}{\gamma_{Av}}\right)-\beta \frac{\theta_1}{\rho }-\left(1-q\right)\beta \left(\frac{1-\xi }{\gamma +\delta }+\frac{\theta \xi}{\delta_A+{\gamma}_A}\right)\right].$$

Solving for *R*_*d*_ = 0 with respect to *q*, we obtain a unique root *q*^∗^given by

$${q}^{\ast }=1-\frac{\beta_v\left(1-\eta \right)\left(\frac{\theta_1}{\rho }+\frac{\left(1-\xi \right)}{\gamma_v+{\delta}_v}+\frac{\theta \xi}{\gamma_{Av}}\right)-\beta \frac{\theta_1}{\rho }}{\beta \left(\frac{1-\xi }{\gamma +\delta }+\frac{\theta \xi}{\delta_A+{\gamma}_A}\right)}\triangleq 1-\frac{\Lambda_1}{\Theta_1}$$

with

$${\Lambda}_1={\beta}_v\left(1-\eta \right)\left(\frac{\theta_1}{\rho }+\frac{\left(1-\xi \right)}{\gamma_v+{\delta}_v}+\frac{\theta \xi}{\gamma_{Av}}\right)-\beta \frac{\theta_1}{\rho },{\Theta}_1=\beta \left(\frac{1-\xi }{\gamma +\delta }+\frac{\theta \xi}{\delta_A+{\gamma}_A}\right).$$

Note that there may never be a level of quarantine rate that is sufficient for *R*_*d*_ = 0.This can happen when

$${\beta}_v\left(1-\eta \right)\left(\frac{\theta_1}{\rho }+\frac{\left(1-\xi \right)}{\gamma_v+{\delta}_v}+\frac{\theta \xi}{\gamma_{Av}}\right)<\beta \frac{\theta_1}{\rho }$$

or

$${\beta}_v\left(1-\eta \right)\left(\frac{\theta_1}{\rho }+\frac{\left(1-\xi \right)}{\gamma_v+{\delta}_v}+\frac{\theta \xi}{\gamma_{Av}}\right)>\beta \left(\frac{\theta_1}{\rho }+\frac{1-\xi }{\gamma +\delta }+\frac{\theta \xi}{\delta_A+{\gamma}_A}\right).$$

Similarly, we can solve for *R*_*d*_ = 0, with respect to *η* and get a unique root *η*^∗^ with

$${\eta}^{\ast }=1-\frac{\beta \left(\frac{\theta_1}{\rho }+\frac{\left(1-\xi \right)\left(1-q\right)}{\gamma +\delta }+\frac{\theta \xi \left(1-q\right)}{\delta_A+{\gamma}_A}\right)}{\beta_v\left(\frac{\theta_1}{\rho }+\frac{\left(1-\xi \right)}{\gamma_v+{\delta}_v}+\frac{\theta \xi}{\gamma_{Av}}\right)}\triangleq 1-\frac{\Lambda_2}{\Theta_2},$$

where

$${\Lambda}_2=\beta \left(\frac{\theta_1}{\rho }+\frac{\left(1-\xi \right)\left(1-q\right)}{\gamma +\delta }+\frac{\theta \xi \left(1-q\right)}{\delta_A+{\gamma}_A}\right),{\Theta}_2={\beta}_v\left(\frac{\theta_1}{\rho }+\frac{\left(1-\xi \right)}{\gamma_v+{\delta}_v}+\frac{\theta \xi}{\gamma_{Av}}\right).$$

Again, we note that *R*_*d*_ =0 will never happen for a vaccine with any efficacy if

$$\beta \left(\frac{\theta_1}{\rho }+\frac{\left(1-\xi \right)\left(1-q\right)}{\gamma +\delta }+\frac{\theta \xi \left(1-q\right)}{\delta_A+{\gamma}_A}\right)>{\beta}_v\left(\frac{\theta_1}{\rho }+\frac{\left(1-\xi \right)}{\gamma_v+{\delta}_v}+\frac{\theta \xi}{\gamma_{Av}}\right).$$

As we mentioned above, vaccination in the population can potentially lead to the increase of transmission effective contacts. One the other hand, those effectively vaccinated will acquire the immunity against the COVID-19 infection. Therefore, the outcome of the transmission in the population is a nonlinear function of the increasing of the effective contacts as a result of introducing a vaccine (or after vaccination), and the effective protection of the vaccine.

**Fig. 2:**
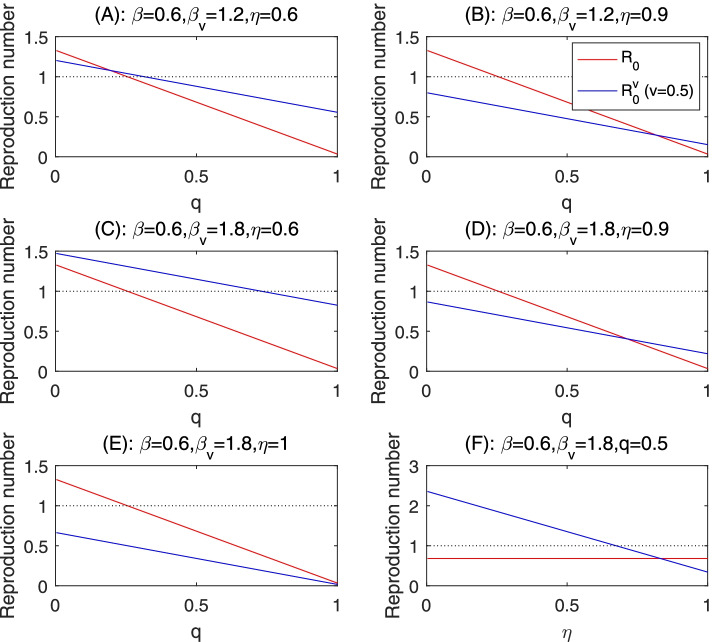
Control reproduction numbers as functions of the quarantine rate *q* or efficacy rate of the vaccine *η* changes, and the critical vaccine efficacy to compensate for the increased transmission effective contacts of vaccines. The other parameters are fixed as those in Table 1

We identified three major scenarios. In Scenario 1, there is a critical value of the quarantine rate above which the vaccination results in higher control reproduction number: ${R}_0^v>{R}_0$ when 1 > *q* > *q*^∗^; and ${R}_0^v<{R}_0$ when 0 < *q* < *q*^∗^. This scenario occurs when the vaccine efficacy rate is moderate so that a reduction of the quarantine rate to the critical value *q*^∗^ will offset the benefit of vaccine. This is shown in Fig. 2(A, B, D), so increasing the effort of contact tracing/quarantine to above the threshold level is more efficient in controlling the spread of COVID-19. In Scenario 2, ${R}_0^v>{R}_0$ for any quarantine rate. This scenario happens when the vaccine efficacy is so low that the control reproduction number when the vaccine is used is always larger than that when vaccine is not used, for any rate of quarantine. This is shown in Fig. 2(C), so an immunization program that leads to substantial increase of the disease transmission effective contacts is counterproductive. In Scenario 3, ${R}_0^v<{R}_0$ for all level of quarantine rate, shown in Fig. 2(E). This is the case with high vaccine efficacy. So an immunization program with small disease transmission effective contacts of vaccines is the most efficient approach to avoid a future outbreak.

We can also view the interplay between the protection of infection through vaccine and the relaxation of social distancing of vaccines by comparing the difference of the control reproduction numbers with and without vaccine when we vary the vaccine efficacy. In the case where 0 < Λ_2_/Θ_2_ < 1 (this happens when the transmission effective contact rate of vaccines is significantly larger than that for non-vaccine), there is a critical vaccine efficacy *η*^∗^ such that ${R}_0^v>{R}_0$ when 0 < *η* < *η*^∗^ and ${R}_0^v<{R}_0$ when 1 > *η* > *η*^∗^, as shown in Fig. 2(F). On the other hand, if Λ_2_/Θ_2_ > 1 then ${R}_0^v<{R}_0$ for all 0 < *η* < 1.

We report some numerical simulations conducted when we fixed the vaccination rate *v* = 0.5, and changed the effective contacts for vaccines *β*_*v*_ (Fig. 2(A-E)) or the efficacy of the vaccine. These numerical simulation results are plotted as the variation of the control reproduction numbers versus the quarantine rate or the vaccine efficacy rate. Recall that the disease spread can be prevented when the control reproduction number is below the threshold 1, marked in the dashed horizontal line. Note that the baseline transmission rate *β* = 0.6, in the reference case of the Province of Ontario, Canada, corresponds to the situation that the social contacts return to the level of 70% of the pre-pandemic normal contacts, while compliance to the personal protection and social distancing measures is high to reduce the transmission probability per contact [28–30]. More precisely, accordingly to the data-driven model-based parameter identification, this corresponds to 1). the level of contacts (4 contacts per day) and transmission probability (0.146 per contact) achieved in stage 3 of social distancing escalation when Ontario closed all non-essential workplace; or 2). The level of contacts (8 contacts per day)—achieved during stage 2 of social distancing escalation (closure of public events and recreational venues, state of emergency) and almost doubling the social distance guideline compliance to reduce the transmission probability to 0.08 per contact.

#### Moderate increase of effective contacts of vaccines

Figure 2(A-B) simulated the situations when the vaccines have the disease transmission effective contacts double those of the non-vaccines. Depending on the vaccine efficacy (*η*), there is always critical value of the quarantine rate below which the control reproduction number with vaccine is higher than that without vaccine. However, the control reproduction numbers, both with and without vaccines, are below the threshold 1, and the outbreak can be prevented when the quarantine rate is higher than 0.25, a level that has been shown to be achievable.

#### Significant increase of effective contacts of vaccines

When the vaccines increase their disease transmission effective contacts to a level so that *β*_*v*_/*β* = 3 in Fig. 2(C-D)), the mass vaccination by a vaccine with low efficacy (*η* = 0.6) will lead the reproduction number consistently higher that that without vaccine for any level of quarantine rate, and the control reproduction number with this vaccine will exceed the threshold value even for a large level of quarantine rate. A combination of lower vaccine efficacy and significant increase of effective contacts of vaccines due to the perceived immunity through vaccine is counterproductive for the prevention and control of the COVID-19 outbreak. We remark that the use of mass vaccination based on high efficacy vaccines, on the other hand, do indeed permit the increase of effective contacts of vaccines so monitoring the efficacy of vaccine against emerging strains is critical.

#### Minimal efficacy to compensate for significant increase of contacts

To illustrate the interplay between the vaccine efficacy and increase of effective contacts of vaccines, we simulated a situation when *β*_*v*_/*β* = 3 and the quarantine rate q=0.5. With this high level of quarantine rate and when the effective contact rate for non-vaccine remains to be *β* = 0.6, we observed that the control reproduction number without vaccine can be reduced to below the threshold 1. However, with 50% of vaccine coverage and while the effective contact rate reaches 1.8, the control reproduction number is above the threshold until the vaccine efficacy reaches 70% (Fig. 2(F)). Increasing the disease transmission effective contacts of the vaccinated population, mass vaccination with low efficacy vaccines will always increase the reproduction number even if the quarantine rate for non-vaccines exposed to the infection is high.

**Fig. 3:**
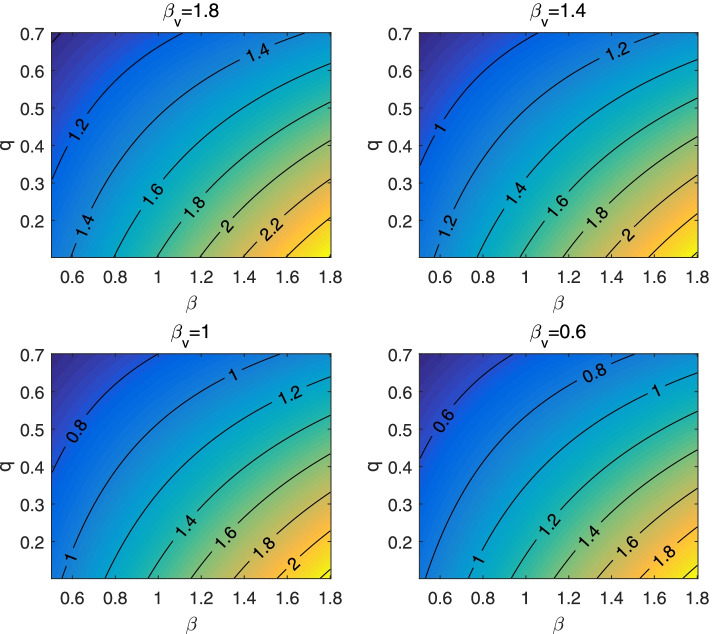
Counter plot of control reproduction number ${R}_0^v$ with respect to transmission rate *β* and quarantine rate *q* for different values of *β*_*v*_. Here, the vaccination coverage is set as *v* = 0.5 while the efficacy rate of the vaccine is assumed to be 0.6 (i.e. *η* = 0.6). The other parameter values are given in Table 1

**Fig. 4:**
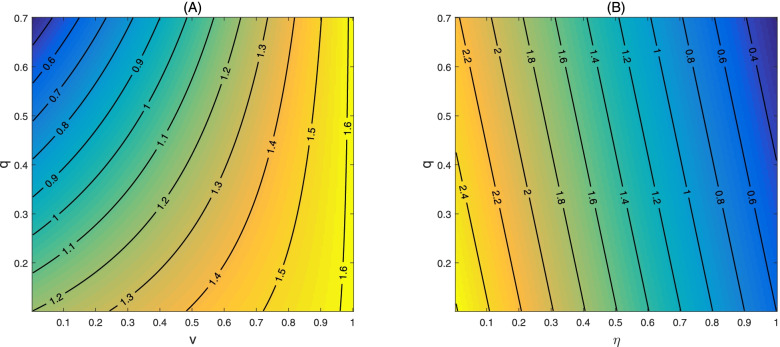
Counter plots of control reproduction number ${R}_0^v$ with respect to vaccination coverage *v* and quarantine rate *q* (**A**); and counter plot of control reproduction number ${R}_0^v$ with respect to vaccine efficacy and the quarantine rate (**B**). The baseline values are fixed as: *β* = 0.6, *β*_*v*_ = 1.8, *v* = 0.5, *η* = 0.6. The other parameter values are given in Table 1

Figure 3 gives the counter plots of the control reproduction number, for different levels of transmission effective contacts of vaccines, as functions of the transmission effective contacts *β* of non-vaccines and the quarantine rate *q*. Comparing the results from four panels of Fig. 3, we found that the control reproduction number increases multiple folds as the transmission effective contacts of vaccines increase. Similarly, we observed that the control reproduction number can be higher when the vaccination coverage increases while other parameter values remain fixed (Fig. 4 A, Fig. 4 B); and that the vaccine efficacy rate is critical for the value of the control reproduction number.

### R2. Impact of vaccination on the final epidemic size

We now focus on quantifying the impact of COVID-19 vaccination on the number of the accumulative infections by the end of an outbreak, i.e. the final epidemic size. We start with the case when there is no vaccination, i.e. *v* = 0. Then model (1) becomes:

2 $$\left\{\begin{array}{c}{S}^{'}=-\frac{\beta \left(I+\theta A+{\theta}_1P\right)S}{N},\kern10.75em \\ {}{L}^{'}=\frac{\beta \left(I+\theta A+{\theta}_1P\right)S}{N}-\sigma L,\kern9.25em \\ {}{P}^{'}=\sigma L-\rho P,\kern13.5em \\ {}{P_Q}^{'}= q\rho P-{\delta}_Q{P}_Q,\kern11.5em \\ {}{I}^{'}=\left(1-\xi \right)\left(1-q\right)\rho P-\gamma I-\delta I,\kern5em \\ {}{A}^{'}=\xi \left(1-q\right)\rho P-{\delta}_AA-{\gamma}_AA,\kern6.25em \\ {}{D}^{'}={\delta}_Q{P}_Q+\delta I+{\delta}_AA-{\gamma}_DD-\alpha D,\kern3.5em \\ {}{R}^{'}=\gamma I+{\gamma}_AA+{\gamma}_DD.\kern10em \end{array}\right.$$

Denote *b*_1_ = (0, *θ*_1_, 0, 1, *θ*, 0), ∏_1_ = (1, 0, 0, 0, 0, 0)^*T*^, *X*_1_ = (*L*, *P*, *P*_*Q*_, *I*, *A*, *D*)^*T*^, and

$${V}_1=\left(\begin{array}{cccccc}\sigma & 0& 0& 0& 0& 0\\ {}-\sigma & \rho & 0& 0& 0& 0\\ {}0& - q\rho & {\delta}_Q& 0& 0& 0\\ {}0& -\left(1-\xi \right)\left(1-q\right)\rho & 0& \delta +\gamma & 0& 0\\ {}0& -\xi \left(1-q\right)\rho & 0& 0& {\delta}_A+{\gamma}_A& 0\\ {}0& 0& -{\delta}_Q& -{\delta}_I& -{\delta}_A& {\gamma}_D+\alpha \end{array}\right),$$

then we have

$${R}_1\triangleq \frac{\beta }{N}{b}_1{V}_1^{-1}{\prod}_1=\frac{\beta }{N}\left[\frac{\theta_1}{\rho }+\frac{\left(1-\xi \right)\left(1-q\right)}{\delta +\gamma }+\frac{\theta \xi \left(1-q\right)}{\delta_A+{\gamma}_A}\right].$$

Let

$${\lambda}_1\left({X}_1\right)\triangleq \frac{\beta }{N}{b}_1{X}_1=\frac{\beta }{N}\left({\theta}_1P+I+\theta A\right),$$

and define a new variable for the weighted sum of diseased components

$${Y}_1\triangleq \frac{1}{R_1}\frac{\beta }{N}{b}_1{V}_1^{-1}{X}_1=\frac{\beta }{R_0}{b}_1{V}_1^{-1}{X}_1=L+P+\frac{\beta }{R_0\left(\delta +\gamma \right)}I+\frac{\theta \beta}{R_0\left({\delta}_A+{\gamma}_A\right)}A$$

as a measure of the epidemic intensity. We calculate that

3 $$\frac{d{Y}_1}{dt}={L}^{'}+{P}^{'}+\frac{\beta }{R_0\left(\delta +\gamma \right)}{I}^{'}+\frac{\theta \beta}{R_0\left({\delta}_A+{\gamma}_A\right)}{A}^{'}=\frac{\beta \left(I+\theta A+{\theta}_1P\right)S}{N}-\frac{\beta }{R_0}\left(I+\theta A+{\theta}_1P\right)={\lambda}_1\left({X}_1\right)\left(S-\frac{N}{R_0}\right).\kern10em$$

Thus we obtain

$$\frac{d{Y}_1}{dS}=\frac{d{Y}_1}{dt}\cdot \frac{dt}{dS}=-{\lambda}_1\left({X}_1\right)\left(S-\frac{N}{R_0}\right)\cdot \frac{N}{\beta \left(I+\theta A+{\theta}_1P\right)S}=-1+\frac{N}{R_0S}.$$

It follows from the above equation that the solution of system (2) satisfies:

4 $$S(t)+{Y}_1(t)-\frac{N}{R_0}\ln S(t)={S}_0+{Y}_1^0-\frac{N}{R_0}\ln {S}_0\ \mathrm{for}\ \mathrm{all}\ t>0.$$

Here,

$${Y}_1^0={L}_0+{P}_0+\frac{\beta }{R_0\left(\delta +\gamma \right)}{I}_0+\frac{\theta \beta}{R_0\left({\delta}_A+{\gamma}_A\right)}{A}_0.$$

We now derive the equation for the final epidemic size of system (2) defined by

*F*_1_ = *S*_0_ − *S*(∞) with $S\left(\infty \right)=\underset{t\to \infty }{\lim }S(t).$ Since *S*^’^(*t*) < 0 for all *t* > 0, we conclude that *S*(∞) exists. Assuming *S*(*t*^∗^) = *N*/*R*_0_, then *S*(*t*) < *N*/*R*_0_ for all *t* > *t*^∗^*.* Then we observe *Y*_1_^’^(*t*) < 0 for all *t* > *t*^∗^, hence $\underset{t\to \infty }{\lim }{Y}_1(t)$ exists. Choosing a sequence *t*_*m*_ → ∞ such that *Y*_1_^’^(*t*_*m*_) → 0 as *m* → ∞, then we have *I*(*t*_*m*_) → 0, *A*(*t*_*m*_) → 0 and *P*(*t*_*m*_) → 0 as *m* → ∞ using equation (3). Therefore, *I*_∞_ = lim inf_*t* → ∞_*I*(*t*) = 0, *A*_∞_ = lim inf_*t* → ∞_*A*(*t*) = 0 and *P*_∞_ = lim inf_*t* → ∞_*P*(*t*) = 0. Based on these, we can choose a sequence *s*_*m*_ → ∞ such that *P*^’^(*s*_*m*_) → 0 and *P*(*s*_*m*_) → *P*_∞_ as *m* → ∞. From the *P* equation in (2) and *P*_∞_ = 0, we obtain *L*(*s*_*m*_) → 0, and accordingly, *L*_∞_ = 0. It follows from (4) *Y*_1∞_ = 0, *Y*_1_(∞) = 0. Taking the limit *t* → ∞ in (4), we have:

$$S\left(\infty \right)+{Y}_1\left(\infty \right)-\frac{N}{R_0}\ln S\left(\infty \right)={S}_0+{Y}_1^0-\frac{N}{R_0}\ln {S}_0.$$

It follows from *S*(∞) = *S*_0_ − *F*_1_ and *Y*_1_(∞) = 0 that the final epidemic size *F*_1_ of system (2) is given by solving

$${F}_1={S}_0-{S}_0{\mathrm{e}}^{-\frac{R_0\left({F}_1+{Y}_1^0\right)}{N}}.$$

If we assume that the vaccination coverage is 100%, that is *v* = 1, then model (1) becomes

5 $$\left\{\begin{array}{c}{V}^{'}=-\frac{\beta_v\left({I}_v+\theta {A}_v+{\theta}_1{P}_v\right)V}{N},\kern3.75em \\ {}{L_v}^{'}=\frac{\beta_v\left({I}_v+\theta {A}_v+{\theta}_1{P}_v\right)V}{N}-{\sigma}_v{L}_v,\kern0.5em \\ {}{P_v}^{'}={\sigma}_v{L}_v-\rho {P}_v,\kern6.5em \\ {}{I_v}^{'}=\left(1-\xi \right)\rho {P}_v-{\gamma}_v{I}_v-{\delta}_v{I}_v,\\ {}{A_v}^{'}=\xi \rho {P}_v-{\gamma}_{Av}{A}_v,\kern5.5em \\ {}{D}^{'}={\delta}_v{I}_v-{\gamma}_DD-\alpha D,\kern3.75em \\ {}{R}^{'}={\gamma}_v{I}_v+{\gamma}_{Av}{A}_v+{\gamma}_DD.\kern2.75em \end{array}\ \right.$$

Similarly, denote *b*_2_ = (0, *θ*_1_, 1, *θ*, 0), ∏_2_ = (1, 0, 0, 0, 0)^*T*^, *X*_2_ = (*L*_*v*_, *P*_*v*_, *I*_*v*_, *A*_*v*_, *D*)^*T*^, and

$${V}_2=\left(\begin{array}{ccccc}\sigma & 0& 0& 0& 0\\ {}-\sigma & \rho & 0& 0& 0\\ {}0& -\left(1-\xi \right)\rho & {\delta}_v+{\gamma}_v& 0& 0\\ {}0& -\xi \rho & 0& {\gamma}_{Av}& 0\\ {}0& 0& -{\delta}_v& 0& {\gamma}_D\end{array}\right).$$

We also have

$${R}_2\triangleq \frac{\beta_v}{N}{b}_2{V}_2^{-1}{\prod}_2=\frac{\beta_v}{N}\left[\frac{\theta_1}{\rho }+\frac{\left(1-\xi \right)}{\delta_v+{\gamma}_v}+\frac{\theta \xi}{\gamma_{Av}}\right]=\frac{R_v}{N\left(1-\eta \right)}$$

and

$${\lambda}_2\left({X}_2\right)\triangleq \frac{\beta_v}{N}{b}_2{X}_2=\frac{\beta_v}{N}\left({\theta}_1{P}_v+{I}_v+\theta {A}_v\right)$$

with

$${R}_v={\beta}_v\left(1-\eta \right)\left(\frac{\theta_1}{\rho }+\frac{\left(1-\xi \right)}{\gamma_v+{\delta}_v}+\frac{\theta \xi}{\gamma_{Av}}\right).$$

Then we can define a new variable, a weighted sum of the disease variables *Y*_2_, as:

$${Y}_2\triangleq \frac{1}{R_2}\frac{\beta_v}{N}{b}_2{V}_2^{-1}{X}_2=\frac{\beta_v}{R_v}\left(1-\eta \right){b}_2{V}_2^{-1}{X}_2={L}_v+{P}_v+\frac{\beta_v\left(1-\eta \right)}{R_v\left({\gamma}_v+{\delta}_v\right)}{I}_v+\frac{\theta {\beta}_v\left(1-\eta \right)}{R_v{\gamma}_{Av}}{A}_v.$$

Taking the derivative of *Y*_2_ with respect to *t*, we obtain

$$\frac{d{Y}_2}{dt}=\frac{\beta_v\left({I}_v+\theta {A}_v+{\theta}_1{P}_v\right)V}{N}-\frac{\beta_v\left(1-\eta \right)}{R_v}\left({I}_v+\theta {A}_v+{\theta}_1{P}_v\right)={\lambda}_2\left({X}_2\right)\left(V-\frac{N\left(1-\eta \right)}{R_v}\right).$$

Therefore,

$$\frac{d{Y}_2}{dV}=\frac{d{Y}_2}{dt}\cdot \frac{dt}{dV}=-1+\frac{N\left(1-\eta \right)}{R_vV}.$$

Then we show that the solution of system (5) satisfies the equation:

6 $$V(t)+{Y}_2(t)-\frac{N\left(1-\eta \right)}{R_v}\ln V(t)={V}_0+{Y}_2^0-\frac{N\left(1-\eta \right)}{R_v}\ln {V}_0\ \mathrm{for}\ \mathrm{all}\ t>0,$$

with

$${Y}_2^0={L}_{v0}+{P}_{v0}+\frac{\beta_v\left(1-\eta \right)}{R_v\left({\gamma}_v+{\delta}_v\right)}{I}_{v0}+\frac{\theta {\beta}_v\left(1-\eta \right)}{R_v{\gamma}_{Av}}{A}_{v0}.$$

We then derive the equation for the final epidemic size of system (5) defined by

$${F}_2={V}_0-V\left(\infty \right)\ \mathrm{with}\kern0.5em V\left(\infty \right)=\underset{t\to \infty }{\lim }V(t).$$

Using the method similarly to what has been developed above, we can show that ${Y}_2\left(\infty \right)=\underset{t\to \infty }{\lim }{Y}_2(t)=0$. Correspondingly, we can take the limit *t* → ∞ in (6) to obtain

$$V\left(\infty \right)+{Y}_2\left(\infty \right)-\frac{N\left(1-\eta \right)}{R_v}\ln V\left(\infty \right)={V}_0+{Y}_2^0-\frac{N\left(1-\eta \right)}{R_v}\ln {V}_0.$$

It follows from *V*(∞) = *V*_0_ − *F*_2_ and *Y*_2_(∞) = 0 that the final epidemic size *F*_2_ of system (5) is given by solving

$${F}_2={V}_0-{V}_0{\mathrm{e}}^{-\frac{R_v\left({F}_2+{Y}_2^0\right)}{N\left(1-\eta \right)}}.$$

Let *L*_0_ = *P*_0_ = *A*_0_ = *L*_*v*0_ = *P*_*v*0_ = *A*_*v*0_ = 0, then

$${Y}_1^0=\frac{\beta }{R_0\left(\delta +\gamma \right)}{I}_0,\kern0.75em {Y}_2^0=\frac{\beta_v}{R_v\left({\delta}_v+{\gamma}_v\right)}{I}_{v0}.$$

Further, normalizing the final size by the total population, we obtain the final disease proportions

$${x}_1=\frac{F_1}{N_0},{x}_2=\frac{F_2}{N_0},$$

and these are obtained by solving

7 $${x}_1=1-{e}^{-{R}_0{x}_1-\frac{\beta }{N_{0\left(\delta +\gamma \right)}}{I}_0},$$

8 $${x}_2=\left(1-\eta \right)\left(1-{e}^{-\frac{R_v{x}_2}{\left(1-\eta \right)}-\frac{\beta_v}{N_0\left({\delta}_v+{\gamma}_v\right)}{I}_0}\right).$$

If *β*_*v*_ = *β*, *δ*_*v*_ = *δ*, *γ*_*v*_ = *γ*, *γ*_*v*_ = *γ*_*Av*_ and *η* = 0, we obtain *R*_0_ < *R*_*v*_. Then, from the above formulas, we have that *x*_1_ < *x*_2_. This inequality remains true if *η* is small due to the continuity. This confirms, from the final epidemic size point of view, that low vaccine efficacy can make the situation worse if we vaccinate against COVID-19. On the other hand, if *η* = 1, then the final epidemic size for model (5) should be 0, i.e. *x*_2_ = 0. Therefore, *x*_1_ > *x*_2_ can happen with high vaccine efficacy rate, so with a high vaccine efficacy, mass vaccination can prevent an outbreak, or mitigate the outbreak (in terms of the final size) if it does happen.

We numerically examined the impact of mass vaccination on the final epidemic size. In our simulations, we fixed the initial total population as *N*(0) = 10000, and the initial population for all the classes of model (1) as:

$$\begin{array}{c}S\left(0\right)=\left(1-v\right)N\left(0\right),L\left(0\right)=0,P\left(0\right)=0,P_Q\left(0\right)=0,I\left(0\right)=0.1,A\left(0\right)=0,\\V\left(0\right)=\left(1-\eta\right)\mathit{vN}\left(0\right),L_v\left(0\right)=0,P_v\left(0\right)=0,I_v\left(0\right)=0,A_v\left(0\right)=0,D\left(0\right)=0,R\left(0\right)=0.\end{array}$$

**Fig. 5:**
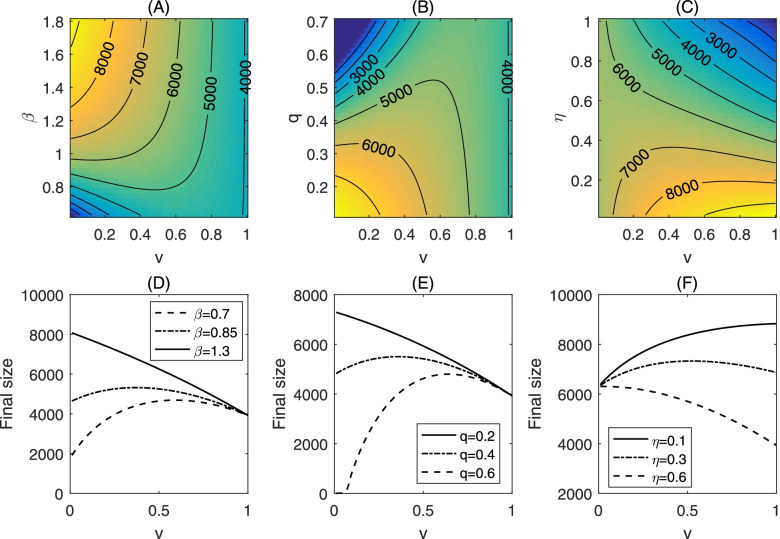
Counter plot of the accumulative cases for model (1) as a function of the transmission effective contact rate and the vaccination coverage in (**A**); as a function of the quarantine rate and vaccination coverage in (**B**); and as a function of the vaccine efficacy rate and vaccination coverage in (**C**). We also plot the accumulative cases as a function of the vaccination coverage, with different transmission efficient contacts (**D**); quarantine rate (**E**) and vaccine efficacy. The baseline values are fixed as: *q* = 0.3, *β* = 1, *β*_*v*_ = 1.8, *v* = 0.5, *η* = 0.6, and the other parameter values are given in Table 1

In Fig. 5, we demonstrate the change of the final epidemic size when we vary the vaccination coverage, vaccine efficacy, quarantine rate and transmission effective contact rate of the infected non-vaccines. A remarkable feature of these plots is the non-monotonic change of the final size when these vaccine characteristics and public health interventions vary. In more details, with a low quarantine rate, the final epidemic size is always decreasing as the vaccination coverage increases. However, the final size changes its monotonicity (from an initial increase to a decrease) as the vaccination coverage increases, if the quarantine rate is high (Fig. 5(B, E)). Similar results can be obtained in terms of the transmission effective contact rate of non-vaccines (Fig. 5(A, D)). This means that with a high level of non-compliance for non-pharmaceutical intervention measures, vaccination against COVID-19 can increase the outbreak final size instead of mitigating the epidemics. It also follows from Fig. 5(C, F) that if the vaccine efficacy is low, a higher vaccination coverage can lead to a lager outbreak.

## Discussion

In this study, we considered the scenario that mass vaccination can potentially lead to an increase of transmission effective contact rate of vaccines and a decrease of their compliance to quarantine/isolation regulation when the vaccine fails to prevent them from acquiring the infection and when they do not present symptoms.

To understand the impact of this non-compliance of vaccines to public health interventions due to the perceived vaccine-provided immunity, we designed a mathematical model to investigate when vaccination in the population is beneficial to controlling of the COVID-19 spread. We addressed this issue, using both the control reproduction number and the final size of epidemic (attack rate).

Our analyses and simulations predict that vaccination in the population affects the control reproduction numbers. Mass vaccination is undesirable if ${R}_0^v>{R}_0$ since the protection offered by the vaccine in the population is offset by the relaxation of social distancing, personal protection and participation in quarantine and isolation if exposed to the infection. Vaccination in the population should also be avoided by all means if ${R}_0^v>1>{R}_0$ since in this case, the use of vaccine with low efficacy in conjunction with relaxation of the non-pharmaceutical interventions will lead to an outbreak that can otherwise be prevented through enhanced non-pharmaceutical interventions. We identified two threshold parameters: the critical quarantine proportion *q*^∗^ (when vaccine efficacy and coverage are fixed) which indicates that a public health contact tracing/quarantine/isolation package with the quarantine proportion higher than *q*^∗^ (if feasible) is more effective than the mass vaccination program; and the minimal vaccine efficacy rate *η*^∗^ (when the quarantine proportion and vaccine coverage are fixed) which indicates that an immunization program with a vaccine efficacy rate below the critical vale *η*^∗^ will not be as efficient as simply investing on the contact tracing, quarantine, and isolation implementation. This conclusion holds under the assumption that vaccines, in comparison with non-vaccines, will have more transmission effective contacts, less personal protection, low compliance to quarantine and/or isolation when individuals are effectively exposed to infection (vaccine failure) and do not display COVID-19 symptoms (pre-symptomatic or asymptomatic infection).

We also numerically and theoretically proved that with a high level of non-pharmaceutical interventions, including the close contact tracing and quarantine, self-isolation and social distancing, vaccination against COVID-19 may boost the outbreak with a bigger final size instead of mitigating the epidemics. Therefore, the minimal efficacy of the vaccine is necessary to compensate for potential increase of transmission contact. This highlights the importance of rapidly evaluating the vaccine efficacy against emerging strains.

**Fig. 6:**
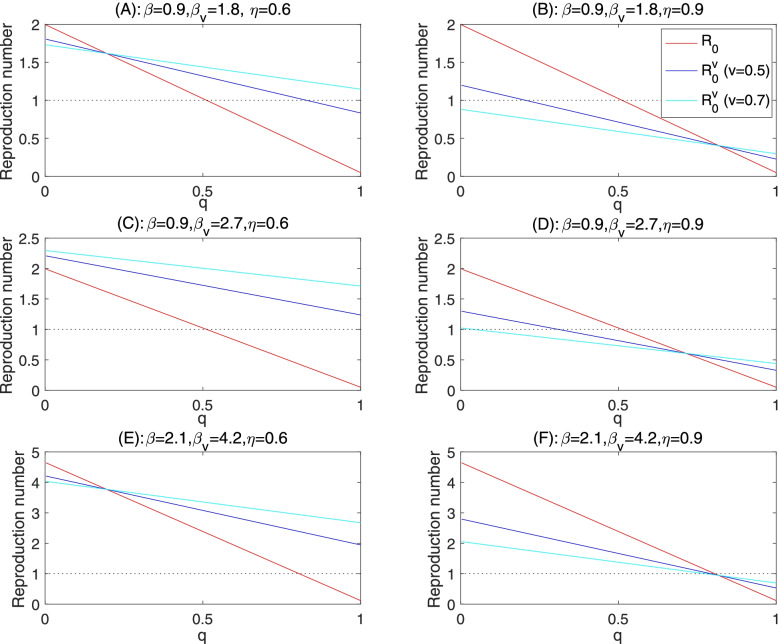
Control reproduction numbers as functions of the quarantine rate *q*, and the critical vaccine efficacy to compensate for the increased transmission effective contacts resulted from the high transmission ability of the mutant strain. The other parameters are fixed as those in Table 1

It is also important to consider emerging mutant strains and their impact on the transmissibility [19, 20]. For illustration, we present here a simulation result that is based on the vaccination rate *v* = 0.5 or *v* = 0.7, and incorporates the increased effective contacts due to a higher transmissibility of the mutant strain (1.5 times of that of the original strain) or the efficacy of the vaccine. We started with the case where the transmission effective contacts of the mutant strain is 1.5 times of the baseline contacts, i.e., *β* = 0.9, and the contacts of vaccines are double (Fig. 6(A-B)) or triple (Fig. 6(C-D)) of those for non-vaccines. Compared with Fig. 2(A-B), we found that if the vaccine efficacy is low (*η* = 0.6), the control reproduction number is an increasing function of the vaccination coverage. In comparison with the case when 50% vaccination coverage is reached, it is still possible to reduce the control reproduction number to below the threshold with a high quarantine rate 0.8, we noted that a vaccine coverage of 70% will result in the situation that the control reproduction number is always above the threshold regardless of the quarantine effort. Therefore, with a mutant strain leading to increase of the transmissibility by 50%, and with a vaccine of low efficacy, the higher vaccination coverage, the higher chance an outbreak will occur.

The situation changes significantly when the vaccine efficacy rate is higher. Indeed, with the efficacy reaching 90%, the control reproduction number will be below the threshold without quarantine (70% coverage) or with very low quarantine rate (0.2), see Fig. 6(B). This is also the case, with *β*_*v*_/*β* = 3 , as shown in Fig. 6(C-D).

We further considered the situation when the effective contacts become higher, *β* = 2.1 and *β*_*v*_/*β* = 2 for the mutant strain, as shown in Fig. 6(E-F) [33, 34]. In this situation, we observed that the quarantine rate and the vaccine efficacy must be significantly high to ensure the control reproduction number below the threshold to avoid an outbreak of COVID-19.

We conclude that a mass vaccination can be successful only when its efficacy is sufficient high. The use of mass vaccination based on a vaccine with relatively low efficacy can be counterproductive if the transmission of effective contacts of vaccines increases. The increase of transmissibility due to mutant strains enforces the need of high efficacy of vaccine and calls for persistence of limiting contacts, continuing personal protection, and contact tracing, quarantine and isolation.

## Data Availability

All data used are publicly available. Professor Dr. Jianhong Wu could be contacted if someone wants to request any type of information or data from this study or any further details.
